# Mechanisms of MHC-I Downregulation and Role in Immunotherapy Response

**DOI:** 10.3389/fimmu.2022.844866

**Published:** 2022-02-28

**Authors:** Brandie C. Taylor, Justin M. Balko

**Affiliations:** ^1^Department of Medicine, Cancer Biology, Vanderbilt University, Nashville, TN, United States; ^2^Department of Medicine, Hematology and Oncology, Vanderbilt University Medical Center, Nashville, TN, United States

**Keywords:** major histocompatibility complex class-I, cancer immunotherapy, immune checkpoint inhibition, antigen presentation, immunoncology

## Abstract

Immunotherapy has become a key therapeutic strategy in the treatment of many cancers. As a result, research efforts have been aimed at understanding mechanisms of resistance to immunotherapy and how anti-tumor immune response can be therapeutically enhanced. It has been shown that tumor cell recognition by the immune system plays a key role in effective response to T cell targeting therapies in patients. One mechanism by which tumor cells can avoid immunosurveillance is through the downregulation of Major Histocompatibility Complex I (MHC-I). Downregulation of MHC-I has been described as a mechanism of intrinsic and acquired resistance to immunotherapy in patients with cancer. Depending on the mechanism, the downregulation of MHC-I can sometimes be therapeutically restored to aid in anti-tumor immunity. In this article, we will review current research in MHC-I downregulation and its impact on immunotherapy response in patients, as well as possible strategies for therapeutic upregulation of MHC-I.

## Introduction

Recent advances in immuno-oncology have shown the benefit of targeting the immune system for the treatment of cancer. Immunotherapies, such as immune checkpoint inhibitors (ICI), have drastically improved the prognosis and overall survival for some cancer patients. However, only a small percentage of patients respond to ICI treatment due to intrinsic or acquired resistance to therapy. Currently, there are few clinically useful biomarkers of response (e.g., Programmed Death-Ligand 1 [PD-L1], tumor mutational burden, microsatellite instability) and limited approved therapies to augment response to ICI. As immunotherapies become more prominent in the treatment of cancer, there is an immense need for understanding the mechanisms of ICI resistance and for the development of new therapeutic strategies to induce long-term responses.

Tumors often adapt various mechanisms to evade immune destruction (1). Some factors that tumors often utilize include immune suppressive mediators (i.e. immune suppressive cytokines, TGF-β), regulatory and suppressive cell (i.e. regulatory T cells), expression of inhibitory molecules (i.e. PD-L1), and defective antigen presentation (2). CD8+ T cells are one of the primary effector cell types that mediate response to ICI and are activated following the recognition of peptide:major histocompatibility complex class I (MHC-I) complexes (pMHC-I) and co-stimulatory signals. T-cell receptors (TCRs) mediate cytotoxic CD8+ T cell activation by binding to their target through interactions with cognate pMHC-I which are ubiquitously expressed by all nucleated cells (3). The efficacy of ICI depends on T cell recognition of pMHC-I on the tumor surface. Activated CD8+ T cells can induce tumor cell death through injecting granzymes and other cytotoxic molecules through perforin-permeabilized membranes at an immunologic synapse. Thus, one mechanism whereby tumors can avoid T cell cytotoxicity is by downregulation of MHC-I. Loss and reduction of MHC-I expression has been correlated with worse overall prognosis and response to ICI in several cancer patient cohorts and clinical trials (4–8). Aberrant MHC-I expression may be reversible or irreversible because of genetic, epigenetic, transcriptional, or post-transcriptional alterations. The reversibility of some mechanisms of MHC-I downregulation creates a potential avenue for therapeutic strategies to restore MHC-I antigen presentation as a possible combinatorial approach with ICI in cancer. This review will address the current known mechanisms of MHC-I downregulation in cancer, implications in patient immunotherapy efficacy, and potential therapeutic MHC-I upregulation strategies to overcome ICI resistance in patients.

## MHC-I Pathway and Regulation

MHC-I is a heterodimer consisting of two domains, a polymorphic human leukocyte antigen (HLA)-encoded heavy α-chain and an invariant light chain termed β2-microglobulin (β2M) (3). There are three classical HLA class I heavy chains, HLA-A, -B, and -C, which are encoded by three separate genes. MHC-I has a high degree of polymorphism derived from each heavy chain’s peptide binding domain. The various HLA alleles have diverse peptide-binding specificities due to the sequence divergence between alleles (9). This diversity of MHC-I allows for a large repertoire of peptides, also known as the immunopeptidome, that can be recognized by CD8+ T cells.

**Figure 1:**
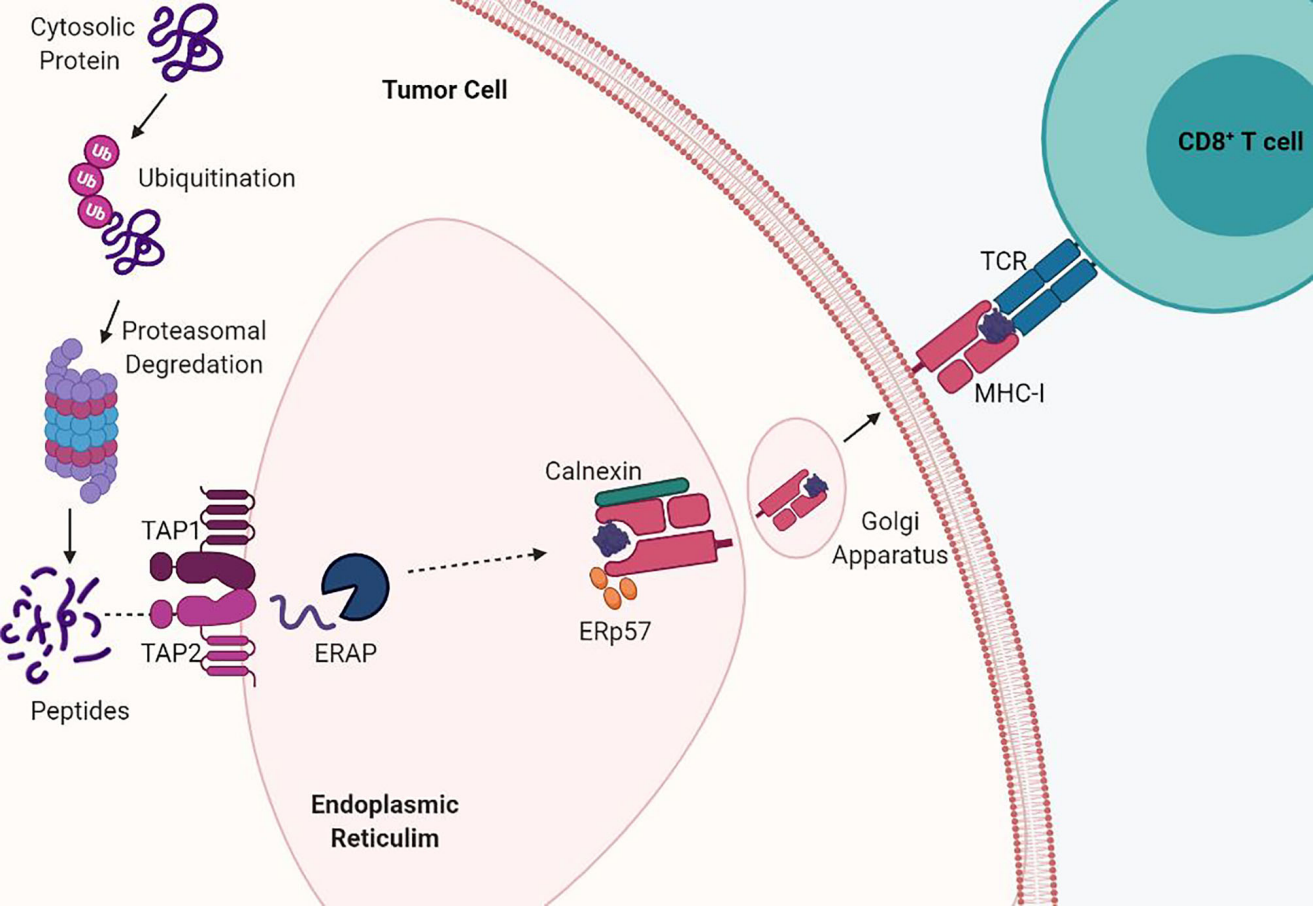
MHC-I antigen processing and presentation pathway. MHC-I presents endogenously derived peptide antigens to CD8+ T cells. Cytosolic proteins are degraded by the proteasome into peptides. These peptides are then shuttled into the endoplasmic reticulum (ER) by TAP transporter proteins. Antigen peptides are then loaded into the assembled MHC-I α-heavy chain and beta-2-microglobulin (β2M) complex and shuttled *via* the Golgi to the cell surface. Acquired mutations in the antigen presentation pathway drive loss of MHC-I.

Antigen presentation by MHC-I begins when endogenous proteins are ubiquitinated for proteasomal fragmentation into peptides (10). The peptides are then translocated from the cytosol into the endoplasmic reticulum (ER) through the transporters associated with antigen processing (TAP1/TAP2) and undergo further processing by ER aminopeptidases (ERAP) (11, 12). Processed peptides of 8 to 10 amino acids in length are required for stabilization of the MHC-I complex and are bound by their side chains through molecular interactions (8). The folding and assembly of the α-heavy chain and β2M is promoted by calnexin and this initial complex is stabilized by the chaperone protein tapasin in complex with ERp57 *via* calreticulin (13). Interaction between TAP1/2 and tapasin permits exchange of peptides into the MHC-I binding groove, release of chaperone proteins, and stabilization of the MHC-I complex. Finally, the peptide:MHC-I complex travels *via* the Golgi apparatus to the cell surface and antigens are presented to CD8+ T cells (**Figure 1**). Cross presentation of exogenous antigens, normally presented by MHC-II on the surface of professional antigen presenting cells, can also be presented through the MHC-I pathway through endocytic mechanisms in which antigens are transported from the endosomal compartment into the cytosol and then degraded by the proteasome and transported into the ER (14). MHC-I expression is induced by both type I and type II interferons and this transcriptional activation is regulated by various conserved cis-regulatory elements at their promoters.

Transcriptional regulation of MHC-I is mediated by three major promoter elements; enhancer A bound by nuclear factor (NF)-κB, interferon-stimulated response element (ISRE) bound by multiple interferon regulatory factor (IRF) family members, and the SYX-module bound by NOD-like receptor CARD domain containing 5 (NLRC5) (15–17). NF-κB induced class-I expression is largely functional at the HLA-A and HLA-B loci, containing NF-κB binding sites at their Enhancer A regions. In contrast, HLA-C and the non-classical alleles are not transactivated by NF-κB (16, 18).

The IRFs are upregulated in response to both type I interferons (IFNs) (e.g., IFNα and IFNβ) and type II IFNs (e.g., IFNγ) and signal through the JAK/STAT pathway (19). Following phosphorylation by JAK, STAT1/STAT2 dimerizes and translocates to the nucleus, where it binds to Gamma-activated sequence (GAS) elements of IFN-stimulated genes, including IRFs, which can subsequently activate the ISRE in the MHC-I promoter (15). It is important to note that studies show that IRF2 can be constitutively expressed and stable in the absence of IFN stimulation (20, 21). Therefore, not only is the IRSE promoter region implicated under inflammatory conditions, but it can also regulate basal MHC-I expression under non-inflammatory conditions. NLRC5 is a critical transcriptional regulator of MHC-I and its associated genes (e.g. β2M, TAP1/TAP2, and LMP2) (22). NLRC5 lacks a DNA-binding domain and is therefore dependent on the enhanceosome to connect to the promoter region of the SYX-module (23, 24). Activation of each of these three promoter regions facilitates antigen presentation by MHC-I on the tumor cell surface and its accessory molecules β2M, TAP, and LMP2.

## Mechanisms of MHC-I Downregulation in Cancer

**Figure 2:**
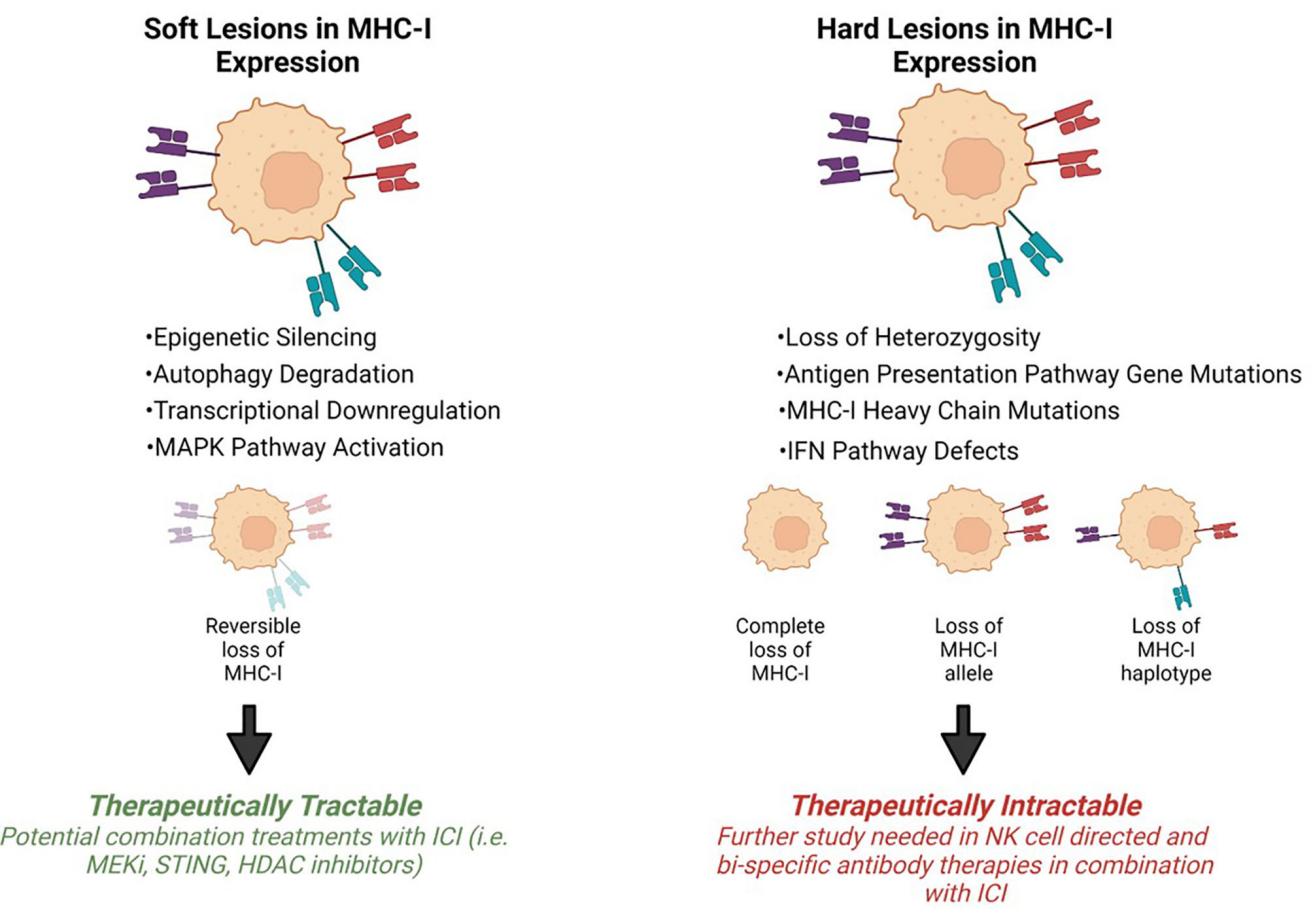
Mechanisms of MHC-I downregulation. Tumor specific MHC-I loss of expression can be classified into soft or hard lesions. MHC-I can be recovered *via* different treatment approaches depending on the type of mutation. Hard lesions, such as *B2m* loss, cannot be therapeutically recovered, however, soft lesions, like epigenetic silencing, can be therapeutically reversed.

Tumor progression is shaped by immunoediting eventually leading to escape from anti-tumor immune responses. Tumor specific MHC-I (tsMHC-I) downregulation has been described in numerous tumor types such as melanoma, breast, colorectal, and cervical cancers (25–28). Loss of tsMHC-I expression is often correlated with shorter overall and progression free survival, and higher instances of metastasis (29–32). The clinical significance of MHC-I is due to the role of antigen presentation and immune control of cancers. Tumors can reduce antigen presentation through several mechanisms including reduced surface expression of tsMHC-I through genetic alterations, antigen depletion, and modulation of transcription. The types of defects that lead to MHC-I disfunction can be divided into two groups: hard lesions (i.e., encoded in the DNA and largely therapeutically intractable) and soft lesions (i.e., those not encoded in the DNA and potentially therapeutically tractable) (**Figure 2**).

### Genetic Defects in the Antigen Presentation Pathway

Defects in the MHC-I antigen presentation pathway are a common mechanism by which tumors can evade the immune response. Reduced levels and complete loss of TAP1/TAP2 expression have been documented in colorectal cancer, renal cell carcinoma, and melanoma resulting in reduced tsMHC-I stability and expression on the tumor surface (33–37). TAP loss has been attributed to a functionally defective allele of Tap1 in numerous solid tumors and cell lines resulting in defective peptide presentation (38). Similarly, studies have shown reduction of tapasin and ERAP have resulted in loss of antigen presentation on the tumor surface (26, 39, 40). Genetic structural alterations of the MHC-I complex are another common finding in tumors that exhibit reduced antigen presentation. Numerous studies document loss of heterozygosity (LOH) associated with chromosome 6p21 resulting in HLA haplotype loss in tumors (41–45). One clinical study designed to test adoptive transfer of T-cells revealed that downregulation of HLA-C was linked with immune evasion and lack of response to treatment (46). Further, a heterozygous deleterious mutation or complete loss of β2M can lead to reduced or absent surface levels of tsMHC-I. Some cancers, such as lymphomas, have high β2M mutation rates in more than 25% of patients (47, 48). Additionally, truncating mutations of β2M were found in metastatic colon and melanoma cancers found to be resistant to the anti-PD-1 therapy, pembrolizumab (25, 49, 50). These observations show that somatic mutations in MHC-I genes are a common mechanism of tumor immune evasion.

### Transcriptional Silencing

The MHC-I pathway can also be dysregulated through the modulation of various transcription factors. NF-κB, bound to the enhancer A region in the MHC-I promoter, is found to be constitutively active in the majority of cancers (28). However, in some cancer types the loss of NF-κB expression results in MHC-I downregulation (21). This suppression of tsMHC-I expression *via* NF-κB can be attributed to multiple proteins, specifically Nedd4 Binding Protein 1 (N4BP1) and tumor necrosis factor (TNF)α-induced protein 3 interacting protein 1 (TNIP1) (51). In addition to NF-κB, IFNs dimerize STAT1 resulting in IRF binding to ISRE therefore playing a large role in MHC-I upregulation during inflammation. Downregulation of both type I and II IFN pathways have been identified as mechanisms leading to resistance to ICI and adoptive T-cell therapies (41, 52, 53). Lastly, the expression of NLRC5, a key regulator of MHC-I transcription, has been found to be correlated with MHC-I expression and patient survival in several cancer types including ovarian, breast, and colorectal cancers as a mechanism of immune evasion (24).

The expression of antigen presentation pathway genes can also be downregulated through epigenetic silencing. This can occur as a result of hypermethylation of the NLRC5 and MHC-I promoter regions (24, 38, 54–57). DNA hypermethylation has shown to be reversible with the treatment of DNA methyltransferase inhibitors (DNMTi) leading to MHC-I upregulation in numerous cancer types (58–63). Another common defect resulting in reduced tsMHC-I antigen presentation is deregulation of histone deacetylases (HDACs), specifically classes I, IIA, and IIB, which remove acetyl groups from histones resulting in a more closed chromatin structure preventing the transcription of MHC-I (59, 64, 65). HDAC inhibitors increase the expression of MHC-I both *in vitro* and *in vivo*. Many of these drugs are already FDA approved (e.g. panobinostat and vorinostat) while others are in clinical trials to determine their efficacy in various types of cancer (62, 64, 66, 67). Overall, these studies reveal the tumor-intrinsic role of epigenetic silencing to evade immune surveillance and the need for further study to understand the functional effect of these mechanisms in cancer.

### Post-Transcriptional Silencing

In addition, loss of MHC-I in cancer can be due to post-transcriptional regulation mediated by non-coding RNAs. MicroRNAs (miRNA), a class of non-coding RNAs, can bind to the 3’ untranslated region (UTR) of mRNAs. Upregulation of some miRNAs have been reported to repress expression of MHC-I in multiple cancers, including melanoma, esophageal carcinoma, and colorectal cancer (68–70). MiRNAs can affect various parts of the antigen presentation pathway, including TAP1/TAP2 and calreticulin (68, 70, 71). For example, TAP2 and MHC-I expression has been shown to be inhibited in esophageal adenocarcinoma through overexpression of miRNA 125a-5p and 148-3p (69). Furthermore, suppression of chaperone proteins by miRNAs (e.g. calnexin, tapasin, and calreticulin) involved in antigen presentation can downregulate MHC-I expression and CD8+ T cell infiltration (70, 72, 73). Taken together, miRNAs can negatively regulate MHC-I through post-transcriptional modification and further study may potentially lead to new therapeutic targets.

## Implications of MHC-I Downregulation in Immunotherapy Response

Recent developments in cancer therapy have shown clear advantages in targeting the immune system. Immunotherapies, such as ICI, have revolutionized the treatment for a range of cancers types, such as melanoma and non-small cell lung cancer (74, 75). ICI increases anti-tumor immunity by blocking intrinsic checkpoints that have an inhibitory role in regulating T cell responses, such as cytotoxic T-lymphocyte antigen 4 (CTLA-4) and programmed cell death 1 (PD-1). Compared to previous standards of care (e.g., chemotherapy and surgery), ICI therapy has significantly improved overall and progression free survival in patients (76).

Despite these successes, ICI treatments still have many limitations and challenges. The benefits of ICI are still very low in some common tumor types, including breast and prostate cancer. ICI therapy is further complicated by observations of intertumoral heterogeneity in the degree of response to therapy. Additionally, a large percentage of cancer patients treated with immunotherapy experience intrinsic or acquired resistance, and many experience immune-related adverse events (77). There is currently a limited number of clinically approved multimodal treatments strategies to improve responsiveness to ICI. Furthermore, there are very few biomarkers that can predict immunotherapy benefit.

Acquired resistance to immunotherapy in several forms of cancer is associated with alterations in the MHC-I antigen presentation pathway. In several studies, downregulation of MHC-I is correlated with resistance to ICIs and adoptive T cell therapy (57, 78, 79). This is presumably mediated by defects in the expression of tsMHC-I for tumor antigen presentation to CD8+ T cells. Analysis of somatic genetic alterations through transcriptomic sequencing and IHC in longitudinal samples of melanoma patients revealed LOH of β2M was found to be a common mechanism of acquired resistance to anti-CTLA and anti-PD-1 therapies (25, 50, 80). Mechanisms of transcriptional loss and evolutionary divergence of the class I heavy chain of specific alleles have been reported in many cancers which results in the loss of MHC-I polymorphism leading to ICI resistance (44, 81, 82). Whole exome sequencing and single-cell transcriptome analysis of colorectal patients resistant to ICI therapy have identified β2M biallelic deletions and truncating mutations in both primary and metastatic tumor lesions (49, 83, 84). Furthermore, a recent study *in vitro* found that β2M suppression in non-small cell lung cancer may be caused by KRAS mutations and STAT5 inhibition (85). Transcriptome and flow cytometric analysis of longitudinal melanoma biopsies collected from patients receiving PD-1 inhibitors identified downregulation of MHC-I to also be associated with TGFβ activity, SNAI1 upregulation, and cancer associated fibroblasts (78). Uncovering additional mechanisms of tsMHC-I and β2M downregulation as potential biomarkers for ICI response and strategies for restoring their expression are areas that merit further study.

Recent studies have demonstrated that in response to anti-PD-1/PD-L1 agents, non-responders have a much higher rate of genomic alterations in the IFNγ pathway as compared to patients that exhibit a clinical benefit. JAK1 or JAK2 loss of function mutations were identified through whole exome sequencing of anti-PD-1 resistant melanoma tumors (25, 86). More specifically, one study determined that JAK1, not JAK2, is the primary mediator of IFNγ induced MHC-I expression and response to anti-PD-L1 therapy (87). Additionally, the transcription factor double homeobox 4 (DUX4) has been shown to block the IFNγ mediated upregulation of tsMHC-I in numerous cancer types *in vitro* resulting in failure to respond to anti-CTLA-4 and anti-PD-1 therapy and decreased overall survival (88). In addition, Ptpn2, was identified through an *in vivo* CRISPR screen to negatively regulate the JAK/STAT and antigen presentation pathways leading to ICI resistance (89). Taken together, these studies suggest that negative regulation of the IFNγ pathway is a mechanism of primary resistance to ICI therapy and supports the design of therapeutic strategies to overcome IFNγ downregulation.

## Therapeutic Strategies to Enhance MHC-I Expression

TsMHC-I associates with improved response to ICI and increased amounts of tumor infiltrating lymphocytes (44, 81, 83, 87, 90)Loss of MHC-I antigen presentation is common in cancers and allows tumors to evade immune surveillance. Although MHC-I loss due to structural genetic alterations such as β2M loss are irreversible, post-transcriptional downregulation of MHC-I can be reversed (**Figure 2**). Therefore, novel strategies to upregulate tsMHC-I are anticipated to improve responses to immunotherapies.

One potential target of upregulating tsMHC-I is through the IFN signaling pathway. IFNγ has been shown to increase expression levels of various antigen presentation genes such as MHC-I and TAP (21, 91). Additionally, NLRC5 has been identified as a key transcriptional coactivator of MHC-I and antigen presentation pathway gene expression (23). Genetic alterations, copy number loss, and somatic mutations of NLRC5 results in reduced MHC-I expression in various cancer types (92). In melanomas derived from patients responding to anti-CTLA-4 and anti-PD-1, there was a significantly higher expression of NLRC5 compared to non-responding patients (57). It has been demonstrated in breast cancer *in vitro* models that NLRC5 was promoted by IFNγ with upregulation of MHC-I suggesting a potential benefit to ICI treatment (93). Although IFN may have a beneficial effect on immunotherapy response, there are limitations to using them in cancer therapy due to potentially life-threatening toxicities seen in patients (94). One possibility of using IFNs for therapeutic upregulation of tsMHC-I without the associated toxicity is through antibody directed, dose-dependent cytokine release at the tumor specific site (95). However, this approach is yet to be tested in large clinical trials.

Additionally, NF-κB pathway activation *via* immune receptors, such as the TNFα receptor, leads to downstream transcription of MHC-I by binding to the enhancer A regulatory element. Stimulation of NF-κB signaling to induce MHC-I upregulation in cancer is a potential therapeutic target. Retinoids, which are clinically used in neuroblastoma through the induction of apoptosis, have been described to induce MHC-I expression through NF-κB signaling (96, 97). Furthermore, in murine models of melanoma, in tumors with deficient IFN signaling MHC-I expression was restored by intratumoral injection of polyinosinic:polycytidylic acid, BO-112 (98). BO-112 restored MHC-I expression through an NF-κB mediated mechanism and independent of NLRC5. A recent study used a CRISPR screening approach to identify factors that decouple the regulation of MHC-I and PD-L1 (99). It was found that depletion of TRAF3 upregulated MHC-I through the NF-κB pathway and TRAF3 small molecule inhibitors increased immuno-sensitivity of cancer cells specifically through MHC-I expression. Stimulation of the NF-κB and IFN-pathways to induce MHC-I expression is further substantiated by studies of Stimulator of Interferon Genes (STING). STING stimulates both NF-κB and IFN-pathways and several STING agonists are being examined in clinical trials in several solid tumors in combination with anti-PD-1 (ClinicalTrials.gov Identifier: NCT04096638, NCT04144140). These studies demonstrate the potential for increasing tsMHC-I expression through transcriptional regulation and cytotoxic CD8+ T cell infiltration to improve patient responses to immunotherapy.

Numerous oncogenic pathways have been reported to affect the expression of MHC-I and related antigen presentation components, including the MAPK and EGFR pathways (100, 101). The MAPK pathway is thought to negatively influence MHC-I expression through decreased IRF and STAT1 expression resulting in reduced levels of tumor infiltrating lymphocytes (100, 102, 103). MEK inhibitors (MEKi), cobimetinib and trametinib, have been shown to enhance IRF1 expression and increased phosphorylation of STAT1 in human epidermal models (104). MEKi in breast cancer and non-small cell lung cancer models *in vivo* and *in vitro* have demonstrated an increase in MHC-I expression and combination of MEKi and ICIs may be a clinically relevant treatment option (79, 103). Overexpression of EGFR has been associated with poor response outcomes to ICI in various cancer models, including non-small cell lung cancer and neuroblastoma (105, 106)Also, the EGFR family member, HER2, was shown to be inversely correlated with MHC-I expression in breast cancer and impairs T cell recognition of peptide: MHC-I complexes (107, 108). Targeting EGFR receptors with antibodies and inhibitors, have resulted in increased expression of MHC-I and antigen presentation components in several cancer cell lines, thus increasing their susceptibility to CD8+ T cell cytotoxicity (109–111). Together, these studies show the importance of targeting oncogenic pathways regulating MHC-I in cancer and the potential for therapeutically restoring anti-tumor immunity and improving immunotherapy response.

A recent study showed that in pancreatic ductal adenocarcinoma (PDAC) MHC-I molecules are selectively targeted for lysosomal degradation through an autophagy-dependent mechanism (112). This study demonstrated that MHC-I is decreased in PDAC through a NBR1-mediated autophagy-lysosomal pathway. It was found that autophagy inhibition using a clinically available antimalarial agent, chloroquine, increased surface levels of MHC-I and anti-tumor T cell responses when combined with ICI therapy (anti-PD-1 and anti-CTLA4). In breast cancer the gene MAL2, which encodes a transmembrane protein associated with protein endocytosis, promotes the turnover of pMHC-I complexes by modulating the interaction between RAB7 and MHC-I (85). MAL2 plays a critical role in the endocytosis-mediated protein degradation of the MHC-I complex and downregulates CD8+ T cell cytotoxicity. Depletion of MAL2 enhanced antigen presentation on tumor cells and activation of CD8+ cytotoxic T cells. Therefore, inhibition of MAL2 is a potentially effective strategy for increasing the efficacy of immunotherapy. Studies have also revealed that high calnexin expression can promote tumor proliferation and growth in lung cancer (113, 114). In both *in vitro* and an *in vivo* melanoma models, Scaffold/Matrix- Associated Region-1 (SMAR1) is directly involved in immune evasion mechanisms by positively regulating MHC-I expression through the downregulation of calnexin (115). Calnexin gene expression was found to be suppressed by the formation of a repression complex that binds to the matrix attachment region (MAR) site on the calnexin promoter. This supports a therapeutic need for small molecule compounds for stabilizing SMAR1 expression, increasing CD8+ cytotoxic T cell infiltration to aid ICI treatment.

Other strategies to upregulate tsMHC-I expression are actively being investigated to ameliorate immunotherapy resistance. Current standards of care in oncology like chemotherapy and radiation are known to induce anti-tumor immune responses without stimulating immunogenic cell death. Studies on various tumor cells *in vitro* have shown that some chemotherapeutic agents augment tsMHC-I expression and *in vivo* further induce tumor CD8+ T cell infiltration. Docetaxel, a common chemotherapeutic agent, can increase expression of components of the antigen presentation pathway, such as TAP and tapasin, without inducing immunogenic cell death *in vitro* (116). Topoisomerase inhibitors (e.g. etoposide) and microtubule stabilizers (e.g. paclitaxel) have also been shown to stimulate MHC-I expression and Treg depletion in murine models (8, 117, 118). These pre-clinical studies indicate a therapeutic advantage to increasing tsMHC-I antigen presentation through combination of ICI and chemotherapeutic agents.

## Potential Therapeutic Solutions for Hard Lesions of MHC-I

As mentioned above, MHC-I downregulation can be categorized into ‘hard’ and ‘soft’ lesions. ‘Hard’ lesions resulting in MHC-I aberrations are irreversible and recovery of these genes is typically only possible by gene editing (5, 119). This has been done through recombinant adenovirus carrying the β2m gene into deficient cells resulting in the induction of MHC-I on the surface (120). Additionally, T-cell bispecific antibodies have been shown to restore cytotoxic T cell activity in tumors that lack MHC-I expression *via* activating immune synapses in cancer cells without dependency on pMHC-I: cognate TCR interactions (121, 122). Additionally in TAP-deficient tumor cells, it a novel class of antigens presented by these cancers were discovered termed T cell epitopes associated with impaired peptide processing (TEIPP) (123). TEIPP targeting CD8+ T cells could be therapeutically utilized for immunotherapy in TAP-deficient tumor cells with low levels of MHC-I (124).

Although downregulation of tsMHC-I allows tumor evasion from T cell mediated cytotoxicity, Natural Killer (NK) cell activation may hold therapeutic premise in heterogenous MHC-I tumors. NK-cells have inhibitory receptors on their surface (e.g. killer cell immunoglobulin-like receptors (KIRs) and MHC-I can function as a ligand to these receptors. Tumors with absent MHC-I expression can be susceptible to NK-cell mediated cytotoxicity because the KIR-mediated inhibitory signaling is lost, a process known as “missing-self recognition” (125). However, tumor cells can evade immunosurveillance through inhibitory cytokines, such as transforming growth factor-β (TGFβ), that suppress NK-cell activity. The plasticity of tumors evading both T- and NK- cell mediated cytotoxicity resulting in tumor escape substantiates the importance of tsMHC-I expression and the key role it plays in patient overall prognosis.

## Conclusion

MHC-I mediated antigen presentation is critical for CD8+ T-cell responses and plays a key role in the adaptive immune response. Although MHC-I is expressed on a wide range of tumor cells, it is often downregulated to evade anti-tumor immunity. There are many mechanisms that can lead to the loss of MHC-I antigen presentation which include defects in the antigen presentation pathway, loss of transcription factors, epigenetic silencing of gene regulatory elements, loss of MHC-I polymorphism, and defects in regulatory signaling pathways. It is important to further understand these mechanisms of MHC-I downregulation and their clinical significance. Furthermore, tsMHC-I has been associated with improved response to anti-PD-1/anti-PD-L1 immunotherapy agents in cancer patients and may be a predictive biomarker in clinical practice. Therefore, identifying novel strategies to upregulate and/or restore tsMHC-I and related pathway components may lead to enhanced anti-tumor response to ICIs.

## Author Contributions

BT and JB contributed to the writing of the final manuscript. All authors contributed to the article and approved the submitted version.

## Funding

Funding for this review was provided by NIC/NCI SPORE 2P50CA098131-17 (JB) and Department of Defense Era of Hope Award BC170037 (JB), the Vanderbilt-Ingram Cancer Center Support Grant P30 CA68485, and the NIH T32 CA009592 (BT).

## Conflict of Interest

The authors declare that the research was conducted in the absence of any commercial or financial relationships that could be construed as a potential conflict of interest.

## Publisher’s Note

All claims expressed in this article are solely those of the authors and do not necessarily represent those of their affiliated organizations, or those of the publisher, the editors and the reviewers. Any product that may be evaluated in this article, or claim that may be made by its manufacturer, is not guaranteed or endorsed by the publisher.
